# A Japanese Patient with Genitopatellar Syndrome Transiently Presenting with Cardiac Intramural Cavity during the Neonatal Period

**DOI:** 10.1155/2020/1731720

**Published:** 2020-08-29

**Authors:** Kiichi Takahashi, Hiroyuki Adachi, Manatomo Toyono, Masato Ito, Akie Kato, Atsuko Noguchi, Tsutomu Takahashi

**Affiliations:** Department of Pediatrics, Akita University Graduate School of Medicine, Akita, Japan

## Abstract

Genitopatellar syndrome (GPS) is a rare autosomal dominant disorder caused by de novo pathogenic variants in the *KAT6B* gene. It is characterized by genital abnormalities, patellar hypoplasia/agenesis, flexion contractures of the hips and knees, corpus callosum agenesis with microcephaly, and hydronephrosis and/or multiple renal cysts. More than half of patients with GPS have congenital heart defects, mostly atrial and/or ventricular septal defects, patent foramen ovale, and patent ductus arteriosus. We report a case of a Japanese neonate with a de novo heterozygous c.3769_3772delTCTA pathogenic variant in the *KAT6B* gene who presented with a cardiac intramural cavity of the ventricular septum at birth. The cavity unexpectedly disappeared at 1 month of age, but trabecular septal thinning and flash remained. The features of the cavity were not consistent with those of congenital ventricular diverticulum or aneurysm, and its identity and prognosis are still unclear. Because patients with GPS may exhibit various forms of cardiac malformation, careful cardiac examination and follow-up are required from birth in cases of suspected GPS.

## 1. Introduction

Genitopatellar syndrome (GPS, OMIM #606170) is a rare syndrome characterized by genital abnormalities, patellar hypoplasia/agenesis, flexion contractures of the hips and knees, corpus callosum agenesis with microcephaly, and hydronephrosis and/or multiple renal cysts [1]. This disorder was first described in 1988 [2], and the term “genitopatellar syndrome” was first used by Cormier-Daire et al. in 2000 [3]. It became evident that GPS is caused by de novo heterozygous truncating pathogenic variants in the *KAT6B* gene in 2012 [4, 5]. *KAT6B* is located at the 10q22.2 region and encodes lysine acetyltransferase 6B, a component of the histone H3 acetyltransferase complex. The *KAT6B* pathogenic variants in the distal and proximal parts of exons 17 and 18 (the last exon), respectively, are known to cause GPS [1, 6]. To date, 18 patients with molecularly confirmed GPS have been reported, and the prevalence of the disorder is estimated to be less than 1 in 1,000,000 individuals [1]. In Japan, only one patient with GPS has been reported [7].

Congenital heart defects are present in more than 50% of patients with GPS [1, 6]. Most of these are atrial and/or ventricular septal defects, patent foramen ovale, and patent ductus arteriosus [1]. Occasionally, tortuous ascending aorta, dilated aortic arch, and pulmonary valve stenosis have been recognized [4]. However, the characteristics and prognosis of cardiac malformations have not yet been fully understood due to the rarity of the disorder. In the present report, we describe a Japanese neonate with GPS presenting with a unique cardiac malformation that had not previously been observed in patients with GPS.

## 2. Case Presentation

The patient is the first son of nonconsanguineous Japanese parents with no family history of congenital anomalies. At the time of his birth, his father and mother were 34 and 25 years old, respectively. Intrauterine growth retardation and hydronephrosis were observed at 22 weeks of gestation, and ventricular enlargement at 36 weeks of gestation was noted via fetal ultrasonography. Amniocentesis revealed a fetal karyotype of 46,XY. The patient was born at 38 weeks and 2 days of gestation by normal vaginal delivery. His birth weight, height, and head circumference were 2,203 g (−1.98 SD), 47.0 cm (−0.67 SD), and 29.8 cm (−2.37 SD), respectively. Apgar scores were 6 at 1 min and 8 at 5 min. He was treated in the neonatal intensive care unit for respiratory distress.

**Figure 1:**
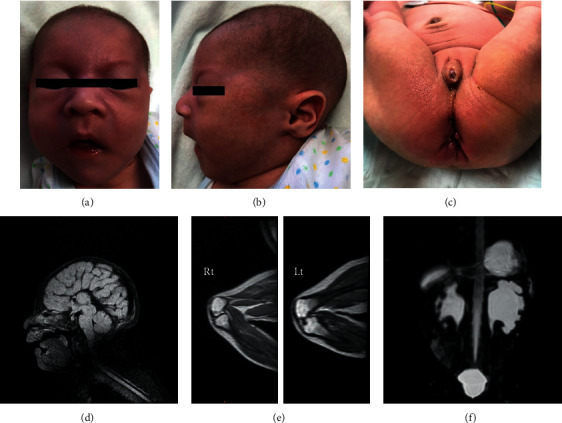
Photographs and MRI of the patient. (a) A front view of the patient showing bitemporal bulbous nose, wide nasal base, and bitemporal narrowing. (b) A side view of the patient showing microcephaly, micrognathia, and retrognathia. (c) Perineum showing hypoplastic scrotum. (d) A sagittal fluid-attenuated inversion recovery (FLAIR) image showing corpus callosum agenesis. (e) Sagittal T2-weighted MRI images of the knees showing absent patellar cartilages. (f) A coronal 3D T2-weighted image showing bilateral renal pelvic dilation.

**Figure 2:**
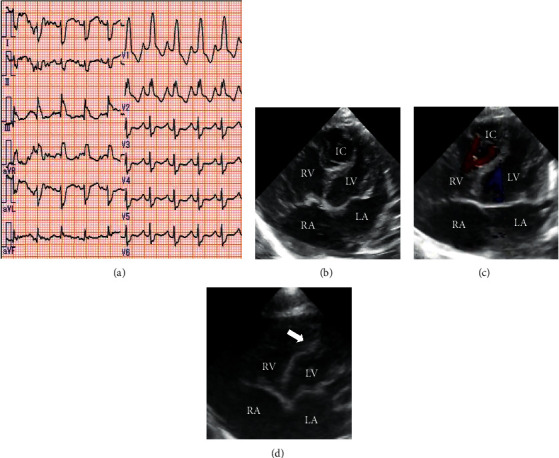
Electrocardiogram (ECG) and echocardiogram of the patient. (a) The 12-lead ECG of the patient showing complete right bundle branch block and right axis deviation. (b) Apical four-chamber view at birth showing the intramural cavity of the trabecular ventricular septum. (c) Doppler ultrasonography at birth showing to-and-fro communication between the right ventricle and cardiac intramural cavity. (d) Apical four-chamber view at 1 month of age showing trabecular septum thinning and flash (white arrow). IC, intramural cavity; RA, right atrium; RV, right ventricle; LA, left atrium; LV, left ventricle.

He had dysmorphic features including microcephaly, bulbous nose, wide nasal base, bitemporal narrowing, micrognathia, and retrognathia (Figures 1(a) and 1(b)). Additionally, limited range of motion in both knees, left clubfoot, and hypoplastic scrotum were recognized (Figure 1(c)), and he had dimples in the bilateral knees. Ultrasonography and magnetic resonance imaging confirmed corpus callosum agenesis, cerebral ventricle dilation, bilateral moderate hydronephrosis, and bilateral missing patellar cartilage (Figures 1(d)–1(f)). Electrocardiogram (ECG) showed sinus rhythm with complete right bundle branch block and right axis deviation (Figure 2(a)). Atrial septal defect, patent ductus arteriosus, moderate tricuspid valve regurgitation associated with pulmonary hypertension, and intramural cavity of the trabecular ventricular septum (13.6 × 11.4 mm) were noted on echocardiogram. Moreover, Doppler ultrasonography depicted three to-and-fro communications (2-3 mm) between the right ventricle and cavity (Figures 2(b) and 2(c)). The cavity wall facing the right ventricle showed septal flash as an abrupt preejection displacement. The intramural cavity disappeared at 1 month of age, but the thinning and flash of the trabecular septum, at which the cavity had existed, remained (Figure 2(d)). He needed tube feeding due to poor oral intake and exhibited severe developmental delay. At 7 months of age, he started using a hearing aid, and when he was 1 year old, he could not hold his head steady.

**Figure 3:**
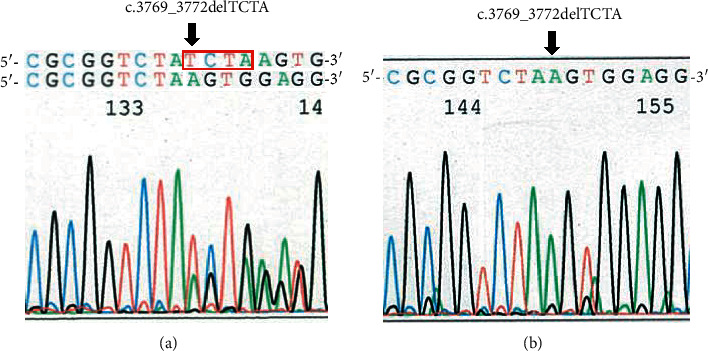
Genomic sequence of the patient. (a) Sanger sequence of *KAT6B* in the patient showing the de novo heterozygous c.3769_3772 deletion in exon 18. (b) Subcloning of mutated alleles of *KAT6B* in the patient showing the de novo heterozygous c.3769_3772 deletion in exon 18.

His clinical symptoms indicated possible GPS. The scientific research of this patient was approved by the Ethical Committee of Akita University (permission number 2013), and written informed consent was obtained from his parents. Subsequently, exons 17 and 18 of *KAT6B* were amplified by polymerase chain reaction (PCR), and Sanger sequencing was performed using a PCR Thermal Cycler Dice Gradient Tp600 (Takara Bio Inc., Shiga, Japan) and an Applied Biosystems 3130 DNA analyzer (Applied Biosystems, Foster City, CA, USA). Mutated and wild-type alleles were separated by subcloning the targeted PCR product into pUC19 DNA (Takara Bio Inc., Shiga, Japan). In exon 18, a de novo 4 bp deletion was detected, c.3769_3772delTCTA, leading to a frameshift and premature stop codon (p.Lys1258Glyfs∗13) (Figures 3(a) and 3(b)).

## 3. Discussion

We encountered a case of GPS with a unique cardiac muscle structure. This malformation showed the following four features: the cavity was noted inside the ventricular septum, the cavity had narrow communication to the right ventricle, the cavity wall showed dyskinesis, and the cavity resolved at 1 month of age. This cavity looked similar to congenital ventricular diverticulum (CVD) or congenital ventricular aneurysm (CVA), but its features were not fully consistent with those of CVD or CVA. Therefore, we named this malformation “cardiac intramural cavity” in the present report. The cardiac intramural cavity, as well as CVD and CVA, has not been described in other patients with GPS.

CVD and CVA, recently often referred to as congenital ventricular outpouchings (CVOs) [8, 9], are generally defined as follows: CVD is a ventricular protrusion that has normal contractility and histologically all three layers of the myocardium, and communication to the ventricular wall of CVD is narrow. In contrast, CVA is a ventricular protrusion that is akinetic or dyskinetic and histologically shows a defect or absence of the myocardium with fibrosis, and communication to the ventricular wall of CVA is broad [9–11]. The etiology of CVD has been considered to be failure of normal embryogenesis, while that of CVA has been considered to be mainly the result of localized ischemia in utero [10, 11]. By definition, the cardiac intramural cavity in the present case was similar to CVA in terms of abnormal contractility; by contrast, it was similar to CVD in terms of the narrow communication to the ventricle. Krasemann et al. reported that the communication width is arbitrary as a diagnostic criterion between CVD and CVA due to the lack of a precise definition of “narrow” versus “broad” [12]. However, Ohlow et al. have recently described that the ratio of the communication width compared to the maximum diameter of the anomaly <1 is CVD and the ratio >1 is CVA [13]. We consider that the communication width in our case was obviously narrow compared to the cavity size.

The location and change in the size of the cardiac intramural cavity are also quite different from those of typical CVOs. Most CVOs are located at the apex or free wall of the ventricle, most frequently on the left ventricular apex [9, 13]. Ohlow et al. reported that CVD and CVA located on the ventricular septum were only 2.5% and 3.2%, respectively, in the analysis of 809 cases of CVD and CVA in the left ventricle [13]. Regarding the change in the size of CVOs, Shuplock et al. have described that among 86 prenatally diagnosed CVOs, only 5 cases resolved before 1 year of age [9]. Although the decrease in the relative size of CVOs in comparison with the growing ventricle may not be rare [14], the complete resolution of CVOs in a short time as our case is considered to be fairly rare. These differences in the features suggest that the cardiac intramural cavity is a different pathological lesion from CVD and CVA. The histological investigation may elucidate the identity of the cavity, but we have not performed myocardial biopsy due to technical difficulties.

The identified c.3769_3772delTCTA pathogenic variant in *KAT6B*, which was demonstrated to produce a truncated protein leading to a gain-of-function effect, has been previously reported in five other patients with GPS [4, 5, 15]. Of these patients, four had congenital heart defects: two had atrial septal defect, one had both atrial and ventricular septal defects [4, 5], and one had a defect type that was not described [15]. These reports suggest that the patients with GPS with c.3769_3772delTCTA pathogenic variant exhibit a typical cardiac phenotype as GPS. However, we could not clarify the particular correlations between the genotype and cardiac phenotype of the cardiac intramural cavity in this report.

After the cardiac intramural cavity disappeared, the thinning and flash of the wall of the trabecular ventricular septum, complete right bundle branch block, and right axis deviation remained in our patient. He has been asymptomatic until now, but his long-term prognosis is still unclear. Similar to CVD and CVA, it may cause cardiac adverse events such as arrhythmia, embolism, ventricular wall rupture, congestive heart failure, and sudden cardiac death [13]. Therefore, we continued careful and frequent follow-up, including ECG and echocardiogram, for our patient. Because patients with GPS may exhibit various forms of cardiac malformation, careful cardiac examination and follow-up are required from birth in cases of suspected GPS. In addition, long-term follow-up and accumulation of additional cases are essential for further understanding of the cardiac characteristics and prognosis of patients with GPS.

In summary, we encountered a case of GPS presenting with a cardiac intramural cavity of the ventricular septum at birth. The features of the cavity were not fully consistent with those of CVD and CVA in terms of the communication width to the ventricle, contractility, location, and change in size. Moreover, the identity and prognosis of the cavity remain unclear. Because patients with GPS may exhibit various forms of cardiac malformation, careful cardiac examination and follow-up are required from birth in cases of suspected GPS.
